# Potential gains in life expectancy by reducing inequality of lifespans in Denmark: an international comparison and cause-of-death analysis

**DOI:** 10.1186/s12889-018-5730-0

**Published:** 2018-07-04

**Authors:** José Manuel Aburto, Maarten Wensink, Alyson van Raalte, Rune Lindahl-Jacobsen

**Affiliations:** 1 Center on Population Dynamics & Department of Public Health, University of Southern Denmark, J.B. Winsløws Vej 9, DK-5000 Odense C, Denmark; 2 Max Planck Institute for Demographic Research, 18057 Rostock, Germany

**Keywords:** Demography, Lifespan variability, Cancer, Mortality, Public health

## Abstract

**Background:**

Reducing lifespan inequality is increasingly recognized as a health policy objective. Whereas lifespan inequality declined with rising longevity in most developed countries, Danish life expectancy stagnated between 1975 and 1995 for females and progressed slowly for males. It is unknown how Danish lifespan inequality changed, which causes of death drove these developments, and where the opportunities for further improvements lie now.

**Methods:**

We present an analytical strategy based on cause-by-age decompositions to simultaneously analyze changes in Danish life expectancy and lifespan inequality from 1960 to 2014, as well as current Swedish-Danish differences.

**Results:**

Stagnation in Danish life expectancy coincided with a shorter period of stagnation in lifespan inequality (1975–1990). The stagnation in life expectancy was mainly driven by increases in cancer and non-infectious respiratory mortality at higher ages (−.63 years) offsetting a reduction in cardiovascular and infant mortality (+ 1.52 years). Lifespan inequality stagnated because most causes of death did not show compression over the time period. Both these observations were consistent with higher smoking-related mortality in Danes born in 1919–1939. After 1995, life expectancy and lifespan equality increased in lockstep, but still lag behind Sweden, mainly due to infant mortality and cancer.

**Conclusions:**

Since 1960, Danish improvements in life expectancy and lifespan equality were halted by smoking-related mortality in those born 1919–1939, while also reductions in old-age cardiovascular mortality held back lifespan equality. The comparison with Sweden suggests that Denmark can reduce inequality in lifespans and increase life expectancy through a consistent policy target: reducing cancer and infant mortality.

**Electronic supplementary material:**

The online version of this article (10.1186/s12889-018-5730-0) contains supplementary material, which is available to authorized users.

## Background

Life expectancy at birth is one of the most commonly used measures of the health status of a population and the performance of the healthcare system [1]. It represents the average age at death if everyone experienced the prevailing death rates throughout their lifetime. Another important dimension is the uncertainty around that expectation (i.e. the variation in ages at death) which is also known as lifespan inequality [2]. Lifespan inequality has become relevant for policy makers with the growing interest in economic and health inequalities, [3, 4] in particular because: [1] it is a marker of heterogeneity in age at death at the macro level, and [2] it is a marker of uncertainty in the timing of death at the micro level [5–7]. Typically, early deaths are more common in underprivileged groups, simultaneously reducing life expectancy and increasing lifespan inequality [8–11]. Both indicators may have implications for individuals’ decisions over their life course. For instance, when to invest in education or when to retire are decisions based on life expectancy but also on the uncertainty surrounding the eventual time of death [10].

Life expectancy is lower in Denmark than in Norway and Sweden for females and males. From 1975 to 1995, while their Scandinavian counterparts showed continuous improvement, life expectancy stagnated among Danish women, while Danish men experienced only slow progress. For both sexes, life expectancy improved after 1995, but remains lower than in Sweden and Norway [12]. Differences between Denmark and Sweden in life expectancy have been thoroughly documented [13, 14]. Among females, the stagnation in life expectancy resulted mainly from the increased mortality of those born from 1919 to 1939, cohorts with high levels of smoking and alcohol consumption compared to their Swedish contemporaries [13, 14]. Similarly, smoking-related mortality was considerably higher in Danish compared to Swedish males because of the widespread use of snus instead of cigarettes in Sweden [15]. While these factors are known contributors to life expectancy differences, [16] their effects on lifespan inequality differences are unknown. Previous evidence has shown mixed results for the effects of smoking on lifespan inequality: little to no effect on the Finnish population, [17] while it increased lifespan inequality in some European countries [18].

The Danish case, juxtaposed with Sweden, is interesting given the shared history, culture and similarities in their healthcare systems [19]. It is unknown how the different age and cause-of-death mortality trends in the two countries would extend to lifespan inequality patterns.

Because life expectancy and lifespan inequality tend to be negatively correlated [5, 7] we hypothesize that 1) during the last decades, Denmark experienced higher lifespan inequality relative to Sweden in females and males; 2) the 1975–1995 stagnation in life expectancy of Danish women was accompanied by stagnation in lifespan inequality; 3) the slow increase in life expectancy for males in the same period was accompanied by slow reduction of lifespan inequality. Because it is well-documented that smoking in the interwar Danish female cohorts was a major cause of the 1975–1995 stagnation in Danish female life expectancy, [14] we hypothesize that 4) any 1975–1995 stagnation in lifespan inequality can be attributed to smoking-related deaths in these cohorts.

Hence, we analyze data since 1960 for Denmark and Sweden to make a cause-by-age analysis of changes in life expectancy and lifespan inequality for both sexes.

## Methods

### Mortality and cause of death data

Period lifetables by sex and single year of age (0–110+) were retrieved from the Human Mortality Database [12] for Denmark and Sweden for the period 1960 to 2014. Cause-of-death data were taken from the WHO Mortality Database to compute the proportion of deaths by cause, age, and sex in a given year [20]. Cause-of-death data are available in 5-year age and single year categories. To increase the accuracy of the resulting estimates, [21] causes of death were ungrouped into single years of age using efficient estimation of smooth distributions [22].

### Cause-of-death classification

Data on causes of death were classified using the seventh, eighth, ninth and tenth revisions of the International Classification of Diseases (ICD) for the period studied [23]. Deaths were grouped into seven major cause-of-death categories aimed at capturing conditions that might have affected mortality in these countries. We considered that smoking prevalence was comparatively high among women (and still remains higher) in Denmark; [14, 24] that the decrease in mortality from heart conditions (cardiovascular revolution) took place during the studied period; [25] and that the management of infectious diseases has improved greatly over the past half century [26]. Hence, we grouped causes of death up to age 84 as follows: 1) Cancers sensitive to smoking, [27] 2) Cancers not sensitive to smoking, 3) Cardiovascular diseases, 4) Non-infectious respiratory diseases, 5) Infectious respiratory diseases, 6) External causes and 7) Rest of causes. For ICD codes and details on the classification see Additional file 1: Table S1. Causes of death above age 85 were not decomposed, because of lower reliability in the presence of multi-morbidities [28]. Our groupings over the various ICD revisions were cross-checked to be consistent with other coding practices across ICD versions in the literature [29]. We also checked for discontinuities in death counts for each of the seven cause-of-death groups over ICD transition years (Additional file 2: Figure S2A and S2B). There were no major breaks at years when ICD versions changed, indicating that cause-specific mortality changes were real and not attributable to inconsistencies in coding practice.

### Lifespan inequality measure

Several dispersion measures have been proposed to analyze lifespan inequality [30]. Here, we use the coefficient of variation (CoV), which is the standard deviation divided by the mean of the lifetable age-at-death distribution, i.e. life expectancy (A See Additional file 1, Section 2 for a brief description). CoV has been found to be a good indicator of lifespan inequality [31]. The strong correlation between dispersion indicators suggests that main conclusions and results would not differ much between measures used [30, 32, 33]. Life expectancy and lifespan inequality (CoV) were calculated for Denmark and Sweden throughout 1960–2014.

A particular attribute of lifespan inequality indicators is the threshold age that separates the ‘young-age component’, also called premature mortality, from the ‘old-age component’ [8]. Saving lives at any age result in increasing life expectancy. For lifespan inequality, improvements below the threshold age decreases inequality, while improvements above increase lifespan inequality.

### Decomposition techniques

Cause-by-age decompositions of the changes in life expectancy and lifespan inequality in Denmark and Sweden were made from 1960 to 2014 using standard decomposition techniques [34]. These decompositions allow us to attribute the age and causes responsible for changes in life expectancy or lifespan inequality between any two periods, for instance between 1975 and 1995.^1^ We quantified the cause-by-age contributions to the current differences in life expectancy and lifespan inequality between Denmark and Sweden for females and males.

## Results

### Trends in lifespan inequality and life expectancy 1960–2014

**Fig. 1:**
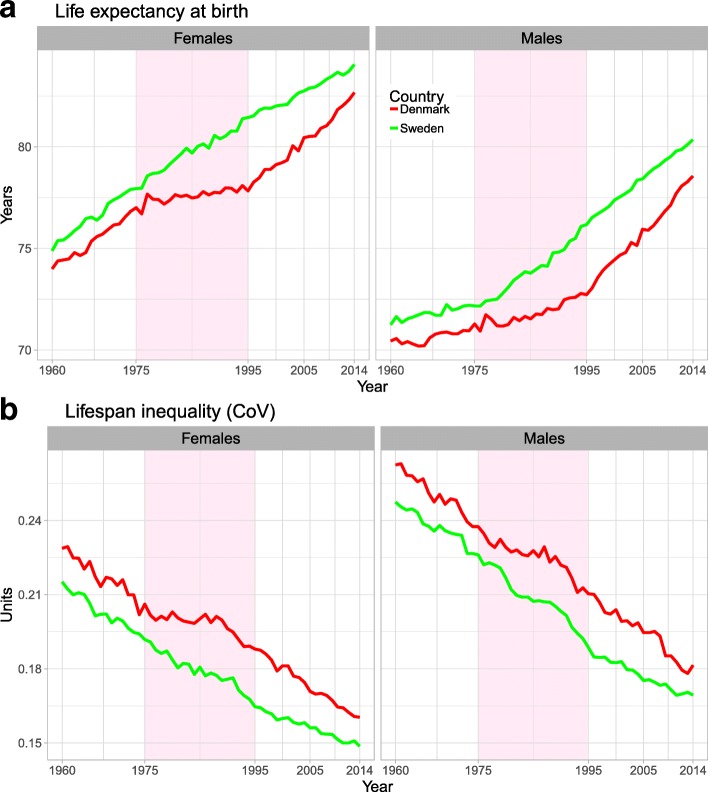
Life expectancy (panel **a**) and lifespan inequality (panel **b**) trends from 1960 to 2014 for Denmark and Sweden by sex. The shaded area refers to the period of life expectancy stagnation in Danish females 1975–1995

The 1975–1995 stagnation in life expectancy for Danish females was accompanied by a shorter period of stagnation in lifespan inequality (Fig. 1a). Swedish females experienced a decrease in inequality and increase in life expectancy throughout the period (Fig. 1a). For males in both countries, life expectancy increase was slow in 1960–1980, but accelerated thereafter, while the decrease in lifespan inequality was more monotonic (Fig. 1b).

### Decomposition of changes in life expectancy and lifespan inequality for Denmark

**Fig. 2:**
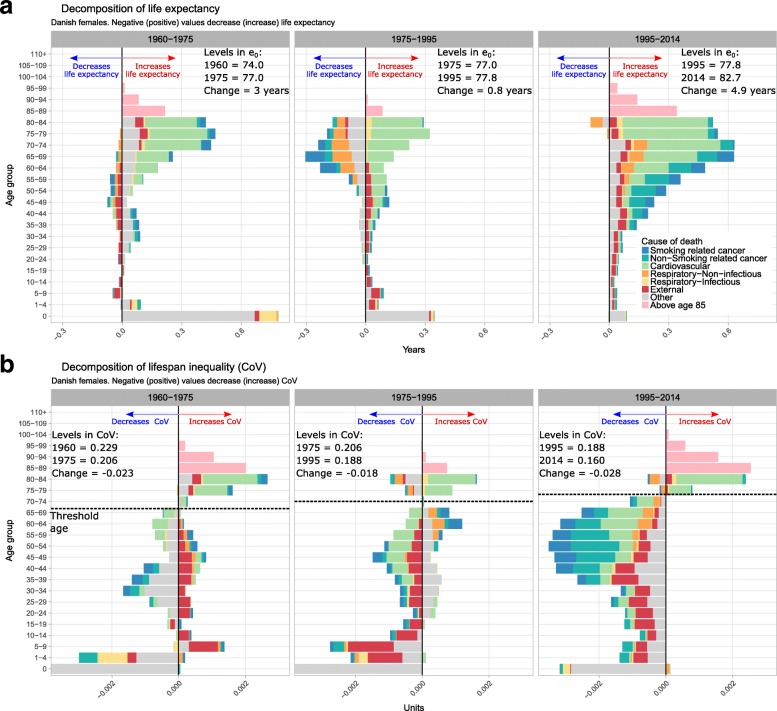
Age and cause contributions to changes in life expectancy (panel **a**) and lifespan inequality (panel **b**) between 1960 and 1975, 1975–1995 and 1995–2014 for Danish females. Note: Age 0 is truncated in panel B since it accounts for the largest contribution

Between 1960 and 1975, Danish female life expectancy increased from 74 to 77 years mainly due to reductions in infant mortality and mid- and old-age cardiovascular mortality (Fig. 2). For males^1^, infant mortality was also reduced, but the contribution from cardiovascular diseases was absent (see Additional file 3: Figure S1), resulting in a small increase in life expectancy from 70.4 to 71.3 years. For both sexes, lifespan inequality was reduced mainly because of the reduction in infant mortality.

Between 1975 and 1995, Danish female life expectancy stagnated at about 77 years because a continued reduction in infant mortality and old-age cardiovascular mortality was offset by an increase in (mainly smoking-related) cancer and non-infectious respiratory mortality between ages 55 and 85 (Fig. 2). Also, reduction in cardiovascular mortality was lower in Danish females relative to Danish males and Swedish females (Additional file 3: Figure S1). The impact of mortality change on lifespan inequality is more complicated due to the presence of the threshold age described earlier: at younger ages mortality reduction results in deaths being compressed into a narrower age range, reducing lifespan inequalities. At older ages mortality reduction stretches out the right tail of the age-at-death distribution, increasing lifespan inequality. Overall, lifespan inequality was mostly unchanged among Danish females because there was little compression of mortality for most causes. Increases in smoking-related cancer and non-infectious respiratory diseases were apparent over both these ‘younger’ and ‘older’ ages with opposite effects, but on balance increased lifespan inequality during the period (Fig. 2). For males, the reduction in lifespan inequality was larger than for females, mainly driven by a reduction in infant mortality and early-life external mortality (Additional file 3: Figure S1).

Between 1995 and 2014, Danish female and male life expectancy increased (from 77.8 to 82.7 and 72.7 to 78.6, respectively) due to almost all causes, particularly cardiovascular conditions which occurred over adult ages. As for lifespan inequality, for both sexes all ages and all causes up to around the life expectancy reduced inequality, while a reduction in cardiovascular mortality at ages higher than life expectancy increased inequality.

### Decomposition of current differences in life expectancy and lifespan inequality between Denmark and Sweden

**Fig. 3:**
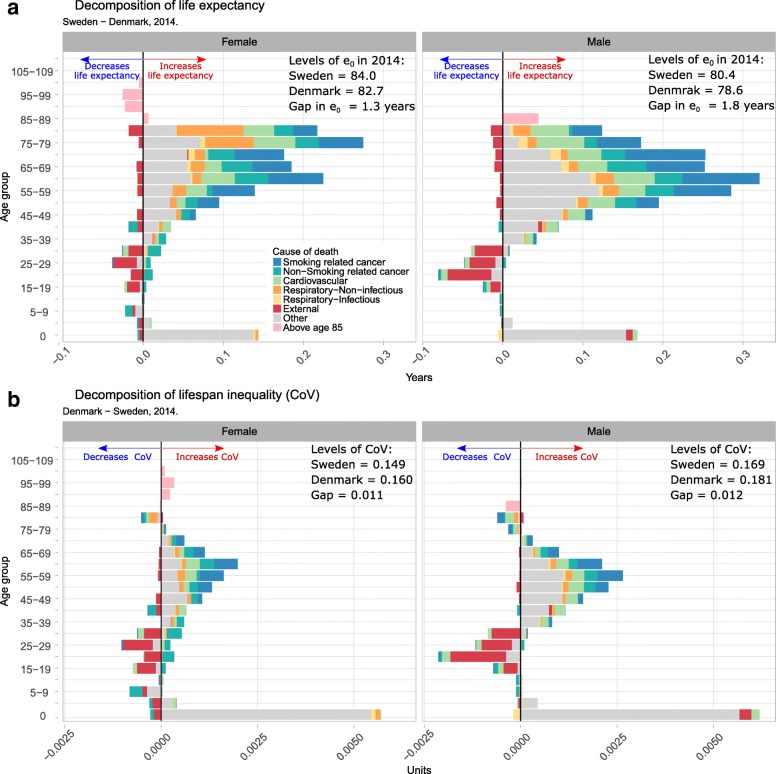
Age and cause contributions to the gap in life expectancy (Panel **a**) and lifespan inequality (Panel **b**) with Sweden in 2014 by sex

Currently (2014),^2^ life expectancy is higher in Sweden than in Denmark for both sexes due to almost all causes at all ages, with the major exception of external mortality being higher in Sweden than in Denmark at all ages, in particular over ages 15–35 (Fig. 3). Two major classes of mortality where Denmark is doing worse than Sweden could be identified. First, infant mortality is higher in Denmark than in Sweden (by a factor two). Second, mid- and old-age cancer mortality is higher in Denmark than in Sweden. Other recent years showed the same pattern.

For lifespan inequality, the same holds: infant mortality and mid-life cancer mortality increase Denmark’s disadvantage relative to Sweden, somewhat offset by lower external mortality between ages 15 and 35 (Fig. 3). However, Denmark’s life expectancy disadvantage relative to Sweden is mainly due to mid- and high-age cancer mortality, while Denmark’s lifespan inequality disadvantage is mainly due to higher infant mortality (Fig. 3).

### Potential gains in Danish life expectancy if lifespan inequality were reduced towards Swedish levels

Reducing mortality from cancers below age 85 would decrease the gap in lifespan inequality by 31 and 22% for females and males, respectively (Table 1). This translates into gains in life expectancy of 0.57 years for females and 0.66 years for males, respectively 44 and 37% of the overall life expectancy gap. Reducing infant mortality (from all causes) to Swedish levels would reduce lifespan inequality by 46% for females and 49% for males. This would be translated into gains in life expectancy of .14 years for females and .16 years for males, respectively 10 and 9% of the total gap.

**Table 1:** Potential gains in life expectancy in Denmark if inequality is reduced (%) to Swedish levels in 2014 by cause of death

Sex		Cause of death category and mortality above age 85	Reduce gap with Sweden in CoV (%)	Reduction in life expectancy gap with Sweden (%)	Potential Gains in life expectancy (years)
Females	1	Smoking-related cancer	18%	25%	0.35
	2	Non-Smoking related cancer	13%	16%	0.22
	3	Cardiovascular	10%	15%	0.21
	4	Respiratory-Infectious	2%	2%	0.03
	5	Respiratory-Non-infectious	7%	17%	0.23
	6	External	−26%^a^	−11%^b^	−0.15
	7	Other	71%	40%	0.55
		Above age 85	5%	−3%^b^	−0.05
Males	1	Smoking-related cancer	15%	26%	0.47
	2	Non-Smoking related cancer	7%	10%	0.19
	3	Cardiovascular	10%	19%	0.33
	4	Respiratory-Infectious	1%	3%	0.05
	5	Respiratory-Non-infectious	5%	7%	0.12
	6	External	−26%^a^	−11%^b^	−0.19
	7	Other	92%	43%	0.77
		Above age 85	0.0	0.0	0.04

^a^Increases the gap with Sweden. Represents potential gains for Sweden if they achieve the levels of Denmark

^b^Increases the gap with Sweden in life expectancy

Note: the sum of percentages differs from 100% due to rounding

Achieving Swedish levels in cardiovascular conditions would decrease the gap in lifespan inequality by almost 10% in both sexes and increase life expectancy by about 3 months. Conversely, if Sweden were to achieve the level of Danish external mortality, it would benefit by two additional months of life expectancy for both sexes. Mortality above age 85 has negligible effect on the difference between Denmark and Sweden in lifespan inequality.

## Discussion

In this study, we found that the same causes and age groups that held back Danish life expectancy in 1975–1995, especially for females, also held back lifespan equality in the same period. Although lifespan inequality has declined and life expectancy has increased since the late 1990s, Denmark still lags its Scandinavian counterparts, despite similarities in social and healthcare systems. The comparison with Sweden suggests that Denmark can now reduce inequality in lifespans and increase life expectancy through the same policy targets: cancer and infant mortality. This suggests an important social development, but also a clear policy target.

Reducing lifespan inequality cannot be the only policy goal, since this would neglect the interests of those who have already lived to higher ages: The effect of mortality reduction on lifespan inequality is large and negative at age zero, decreases with increasing age, and reverses at a unique threshold age, so that mortality reductions above this threshold age increase lifespan inequality [5, 35]. Therefore, the causes that extend average lifespan and the causes that reduce lifespan inequality are not necessarily the same [36]. Smoking-related mortality is a clear example of this. In Denmark, life expectancy stagnated over the 1975–1995 period because mortality reduction from most causes of death was offset by mortality increase from smoking-related causes. These increases in smoking-related mortality had a smaller net impact on lifespan inequality compared to life expectancy over the same period, since smoking-related mortality occurred both below and above the threshold age. By the latest period 1995–2014, however, reduction in smoking-related mortality was comparatively more important for decreases in lifespan inequality (19.4%) than increases in life expectancy (11.2%). In general, the impact of smoking on lifespan inequality is dependent on both the age of smokers compared to non-smokers (the maturity of the smoking epidemic), as well as the actual impact of smoking on mortality at different ages [17]. Similar to what was found in a comparison of G7 countries, [36] reductions in injuries and child mortality were relatively more important for lifespan inequality decrease than for life expectancy increase.

In the 1975–1995 period, non-smoking cancers also contributed (albeit to a small extent) to reductions in life expectancy and increases in lifespan inequality. The conservative definition of smoking-related cancers in this paper is one explanation for this phenomenon. Competing risks is another: people who previously died of other causes could die of cancer, and these increased cancer rates would show up as holding back life expectancy. In this respect, we note that non-smoking related cancer was on the rise also in Sweden, so it is likely not a phenomenon specific to Denmark. Specifically for Danish females, other risk-taking behavior may have led to increased cancer rates in general [13, 14].

Causes of death that drive within-country changes in lifespan inequality are not necessarily the same as the causes of death that drive contemporary gaps between countries [37]. However, the comparison with Sweden suggests that Denmark can simultaneous increase life expectancy and decrease lifespan inequality by targeting two main causes of death: cancer and infant mortality. Reducing lifespan inequality towards Sweden by these conditions would lead to an increase of 0.7 and 0.8 years in life expectancy for females and males in Denmark, respectively. To put this in perspective, in 2014 the infant mortality rate in Denmark is twice as high as in Sweden, which is one of the lowest among developed countries [12]. Although mortality at very young ages may be affected by different registration practices in high income countries (e.g. non-viable live births registered as stillbirths), [38] the Nordic countries do not show evidence of such patterns [39]. Moreover, even after controlling for gestational age Sweden showed lower infant mortality rates than Denmark [40]. Thus, infant mortality is the largest single contributor to the gap with Sweden in terms of lifespan inequality. Preventive policies focusing on prenatal risk factors and improving maternal health before and during pregnancy, [41] as well as efforts to reduce the risk of sudden infant death syndrome [42] could help to reduce infant mortality towards Swedish levels.

Targeting cancer is another clear public health intervention to reduce lifespan inequality and increase life expectancy in Denmark, confirming the priority given to this objective for the last two decades through the National Cancer Plans [43]. Our results show that improvements in cancer mortality have had an effect on both health indicators over the last 20 years. However, Denmark had the highest mortality rates from all neoplasms in the European region, and the female population exhibited the highest lung cancer mortality rates [24]. This is in line with our comparison with Sweden and with previous evidence highlighting the role of smoking behaviors on life expectancy trends [14].

For Sweden, the decomposition results suggest that young-age external mortality can be further reduced. According to the WHO, males in Denmark have lower age-standardized external mortality rates (39 per 100,000) than Sweden and Norway (50.6 and 52 respectively) in 2014 [44]. Our results further show that these differences are concentrated between ages 15 and 40. Moreover, since the late 1990s, Swedish and Norwegian males have experienced higher suicide rates between ages 15 and 24 [45].

The mere observation that Sweden is doing better than Denmark for most causes of death does not mean that Denmark could easily do better. However, it does provide a starting point for public health intervention. For instance, previous evidence suggests that focusing on vulnerable and less socially advantaged subgroups may reduce suicide rates among the young [45, 46].

For other countries that lag a comparable country, similar decompositions can be made. This may not result in a clear and consistent message: causes of death that hold back life expectancy may not be the same as the causes of death that hold back equality. Yet if it does, as in the case of Denmark when compared to Sweden, the benefits are substantial, because the policy goals can be so clearly stated. We therefore suggest that this method could be a valuable tool for epidemiologists and policy makers alike.

As any cause of death analysis, our study has the limitations that: 1) causes of death are treated as mutually exclusive, while they may not be (e.g., poor sight due to diabetes may lead to an accident); 2) medical doctors and even coroners have imperfect knowledge about causes of death; and 3) trends in awareness of certain diseases and changing insights in disease processes affect classification. Yet through using otherwise high-quality data and broad categories of causes of death, we believe we have achieved a useful, workable grouping of causes of death. In addition, we performed a sensitivity analysis to assure consistency of grouping across ICD versions and did not find significant variation when ICD revisions changed (Additional file 2: Figure S2). In addition, although the correlation between lifespan indicators suggest that our results would not differ had we used a different indicator, relative inequality indicators (e.g. coefficient of variation) differ in properties from indicators that measure absolute lifespan inequality (e.g. standard deviation). To alleviate any concern we replicated our results using the standard deviation (Additional file 4: Figure S3, Additional file 5: Figure S4, Additional file 6: Figure S5, Additional file 7: Figure S6) and did not find major differences.

Lifespan inequality is an important dimension of population health. By looking at this dimension we could disclose how lifespans differ within Denmark and Sweden. Moreover, our decomposition by age and cause of death allowed us to identify conditions and ages that contribute the most to lifespan inequality changes, and we were able to translate them into potential gains in life expectancy if efforts were concentrated in these ages and causes of death.

## Conclusions

Lifespan inequality together with life expectancy gives a broader perspective on the effect of mortality changes on population health. Our results show that life expectancy and lifespan inequality have been negatively correlated since at least 1960 in Denmark. Currently, Denmark lags Sweden both in terms of high life expectancy and low inequality due to two main causes: infant mortality and cancer. Denmark therefore has a clear and consistent public health policy target: reduce infant mortality and cancer mortality. Our approach demonstrates how reduction in lifespan inequality as a policy target can be translated into gains in life expectancy.

## Additional files

Additional file 1: Details on the classification, ICD codes for the cause-of-death classification and brief description of the indicator of lifespan inequality. (PDF 263 kb)

Additional file 2: **Figure S2A and S2B.** Death counts by cause-of-death group for Denmark (A) and Sweden (B). Colored-vertical lines indicate changes in ICD versions. For example, in the case of Denmark, the green vertical line indicates the change from ICD 7 to ICD 8, which was in 1969. (ZIP 269 kb)

Additional file 3: **Figure S1.** Age and cause contributions to changes in life expectancy (panel A) and lifespan inequality (panel B) between 1960 and 1975, 1975–1995 and 1995–2014 for Danish males. Note: Age 0 is truncated in panel B since it accounts for the largest contribution. (PDF 126 kb)

Additional file 4: **Figure S3.** Trends in the standard deviation for Sweden (green) and Denmark (red). (PDF 6 kb)

Additional file 5: **Figure S4.** Age and cause-decomposition of the change in the standard deviation over time for Danish females. Note: the age zero is truncated for visualization purposes. (PDF 9 kb)

Additional file 6: **Figure S5.** Age and cause-decomposition of the change in the standard deviation over time for Danish males. Note: the age zero is truncated for visualization purposes. (PDF 9 kb)

Additional file 7: **Figure S6.** Age and cause-decomposition of the difference in the standard deviation between Denmark and Sweden 2014. (PDF 46 kb)

## Abbreviations

CoV: Coefficient of variation
ICD: International Classifications of Diseases

## Footnotes

1 We have created an interactive app where the reader can analyze any period he/she might be interested in for any sex. Available online app.

2 Results for any year from 1960 to 2014 and for Denmark vs Sweden available in online app.
