# Supplementary material for: Potential gains in life expectancy by reducing inequality of lifespans in Denmark: an international comparison and cause-of-death analysis
Source: BMC Public Health. 2018 Jul 4;18:831. doi: 10.1186/s12889-018-5730-0 (PMC6033219; doi:10.1186/s12889-018-5730-0)
Supplement: Supplementary file 1 — Details on the classification, ICD codes for the cause-of-death classification and brief description of the indicator of lifespan inequality. (PDF 263 kb) [file 12889_2018_5730_MOESM1_ESM.pdf]

**Additional file to: Potential gains in life expectancy by reducing inequality of lifespans in Denmark: An international comparison and cause-of-death analysis.**

**Authors:** José Manuel Aburto [a,b*], Maarten Wensink [a*], Alyson van Raalte [b] & Rune Lindahl-Jacobsen [a]

**Author affiliations:**

[a] Institute of Public Health- Unit of Biodemography, University of Southern Denmark, Odense 5000, Denmark.

[b] Max Planck Institute for Demographic Research, Rostock 18057, Germany

* These authors contributed equally to the paper

**Corresponding authors:** JMA

Institute of Public Health- Unit of Biodemography, University of Southern Denmark

J.B. Winsløws Vej 9, DK-5000 Odense C

T: +45 65509416

jmaburto@health.sdu.dk

**Table S1. ICD code for the cause-of-death classification.**

| Cause of Death | ICD-7 | ICD-8 | ICD-9 (Sweden) | ICD-10 |
|---|---|---|---|---|
| Cancer, smoking-related | A044-A050, A052, 157, 180-181 | A045-A051, A055, 157, 188-189 | B08, B090-B094, B096, B100-B101, 180, 188-189 | C00-C21, C25, C30-C34, C53, C64-C68 |
| Cancer, not smoking-related | A051, A053-A056, A058-A059, 155-156, 158-160, 164-165, 175-176, 178-179, 192-195, 198-199 | A052-A054, A056-A057, A059-A60, 155-156, 158-160, 163, 171, 183-184, 186-187, 190-199 | B095, B099, B109, B11, B13-B14, 179, 181-187 | C22-C24, C26, C37-C39, C40-C41, C43-C52, C54-C58, C60-C63, C69-C97 |
| Cardiovascular | A063, A070, A079-A086 | A064, A080-A088 | B181, B25-B30 | E10-E14, I00-I99 |
| Respiratory, infectious | A087-A092, A095 | A089-A092, A095 | B310-B312, B320-B322 | J00-J06, J09-J18, J20-J22, J34.0, J36, J39.0, J39.1, J85, J86 |
| Respiratory, non-infectious | A093, A094, A096, A097 | A093, A094, A096 | B313-B315, B319, B323-B327, B329 | J30-J33, J34.1-J34.3, J34.8, J35, J37, J38, J39.2, J39.3, J39.8, J39.9, J40-J47, J60-J70, J80-J82, J840-J841, J848-J849, J90-J99 |
| External | A138-A150 | A138-A150 | B47-B56 | S00-T89, V01-Y84 |
| Other | A001-A043, A060-A062, A064-A069, A071-A078, A098-A137 | A001-A044, A061-A063, A065-A079, A097-A137 | B01-B07, B184-B185, B15-B17, B180, B182-B183, B189, B19-B23, B33-B46 | A00-B89, B99, D00-D48, D50-D89, E00-E07, E15-E16, E20-E35, E40-E46, E50-E68, E70-E90, F00-F99, G00-G99, H00-H59, H60-H95, K00-K93, L00-L99, M00-M99, N00-N99, O00-O99, P00-P96, Q00-Q99, R00-R99 |

## 1. Details on the classification

Primary malignancies that are sensitive to smoking are found predominantly in the respiratory, digestive and genitourinary tracts, in line with the principle that where smoke or its products pass, the risk of cancer rises. Primary malignancies in the gastrointestinal tract from mouth to anus were classified as sensitive to smoking, with the exception of liver cancer, for which detail could not be reconstructed across ICD versions (see below). Cancer in the respiratory tract was also classified as sensitive to smoking. In addition, it has been proven that smoking causes cancer of the uterine cervix, the ovaries (mucinous carcinoma), the bladder, the kidney (pelvis and body) and the ureter. For mucinous carcinoma of the ovaries, detail could not be reconstructed across ICD versions (see below). Malignancies in the urinary tract were classified as being sensitive to smoking.

The resolution of the ICD classification has grown substantially over the years. As we analyzed deaths from 1960 through 2014, we used ICD-7 through ICD-10. The specifically identified categories "cancer sensitive to smoking" and "respiratory infectious", are based on the smallest common denominator: only if a specific disease could be separately identified across ICD versions did we include it in these groups. For instance, myeloid leukemia is associated with smoking, but ICD-7 and -8 contain only a category 'leukemia', without subclassification. Hence, for reasons of consistency across classifications, myeloid leukemia is considered as not sensitive to smoking throughout. Also, ICD-7 and ICD-8 have an overall rest group for malignant neoplasms, while ICD-9 and ICD-10 have rest groups for each tract, if known. Because ICD-7 and ICD-8 do not have these detailed rest groups, rest groups were classified as not sensitive to smoking for all ICDs.

## 2. Brief description of the indicator

In lifetable notation, it is:

$$CoV_a = \frac{\sqrt{\int_a^\omega (x - e_a)^2 f(x)\mathrm{d}x}}{\int_a^\omega \ell(x)\mathrm{d}x} = \frac{\sigma_a}{e_a}. \qquad (1)$$

Where $a, e_a, \sigma_a$ and $\omega$ denote the starting age at death of the density function, life expectancy at age $a$, the standard deviation at age $a$, and the open-aged interval (110+ in our case), respectively. We study the coefficient of variation from birth, i.e. $a = 0$.
