# Supplementary figures and images for: Potential gains in life expectancy by reducing inequality of lifespans in Denmark: an international comparison and cause-of-death analysis
Source: BMC Public Health. 2018 Jul 4;18:831. doi: 10.1186/s12889-018-5730-0 (PMC6033219; doi:10.1186/s12889-018-5730-0)

# A) Denmark

Years of change in ICDs: 1969 and 1994

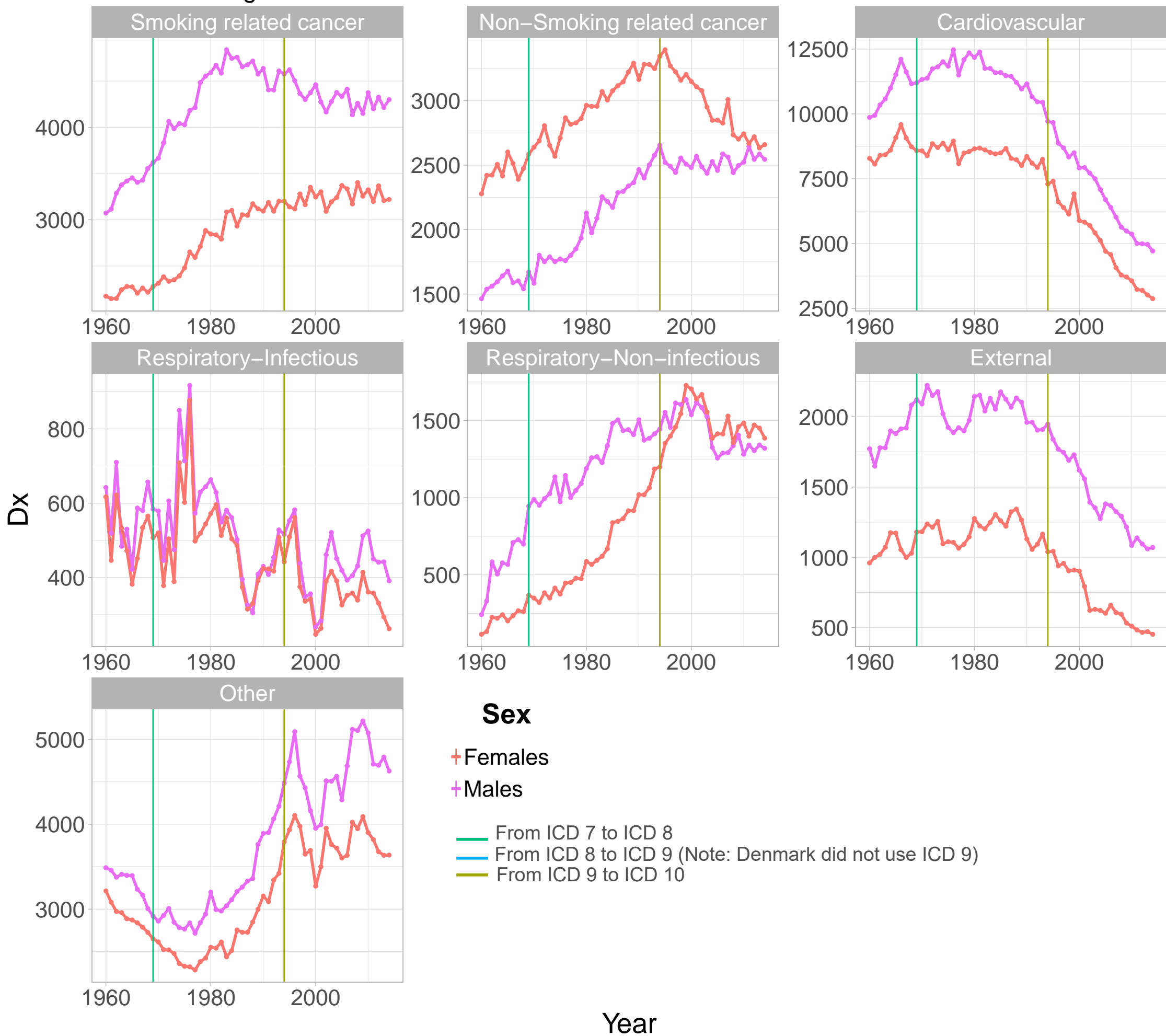

Supplement: Supplementary file 2 — Figure S2A and S2B. Death counts by cause-of-death group for Denmark (A) and Sweden (B). Colored-vertical lines indicate changes in ICD versions. For example, in the case of Denmark, the green vertical line indicates the change from ICD 7 to ICD 8, which was in 1969. (ZIP 269 kb) [file 12889_2018_5730_MOESM2_ESM.zip › Appendix_Fig2AR2.pdf]

# B) Sweden

Years of change in ICDs: 1969, 1987 and 1997

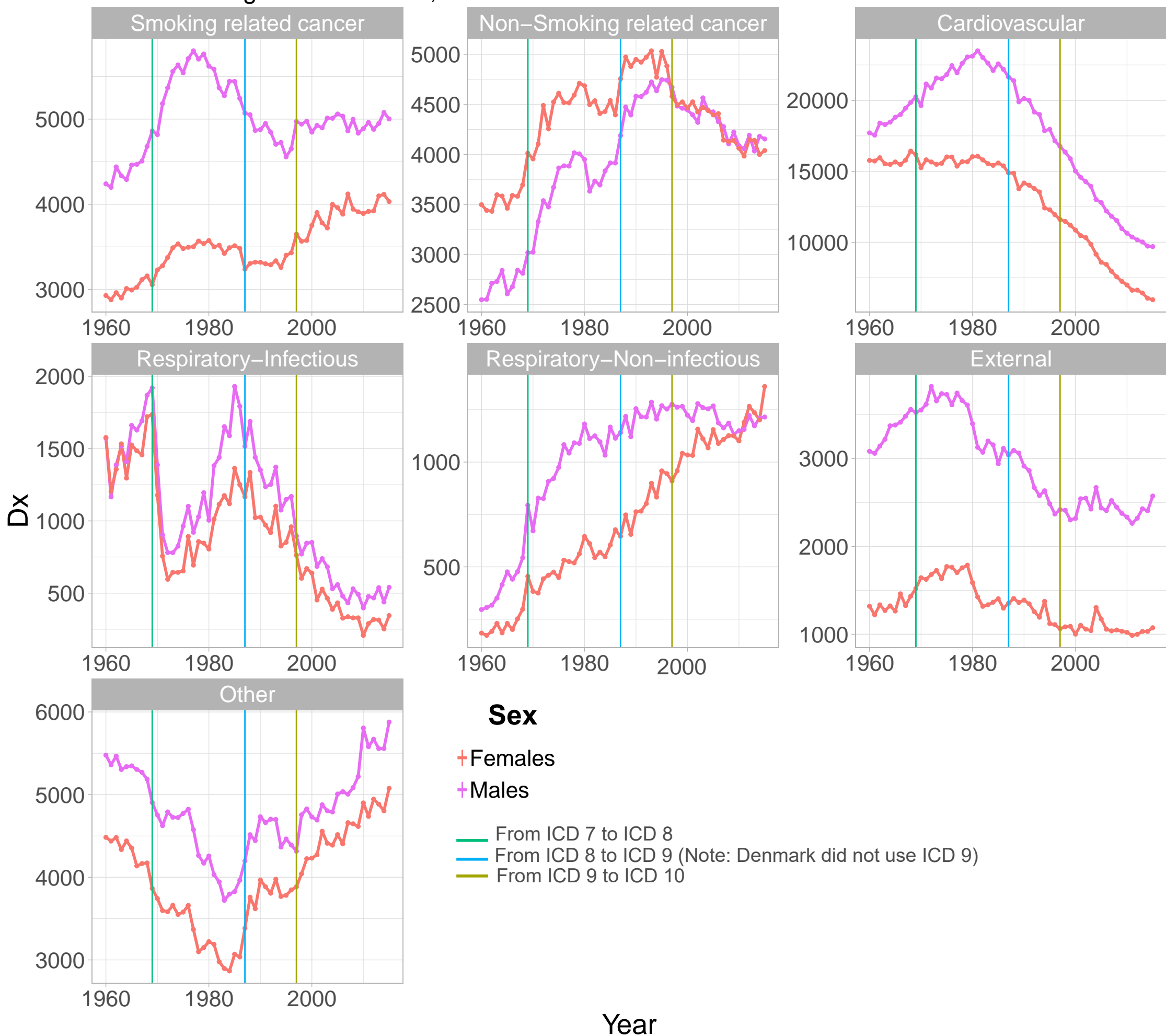

Supplement: Supplementary file 2 — Figure S2A and S2B. Death counts by cause-of-death group for Denmark (A) and Sweden (B). Colored-vertical lines indicate changes in ICD versions. For example, in the case of Denmark, the green vertical line indicates the change from ICD 7 to ICD 8, which was in 1969. (ZIP 269 kb) [file 12889_2018_5730_MOESM2_ESM.zip › Appendix_Fig2BR2.pdf]

# Trends in standard deviation

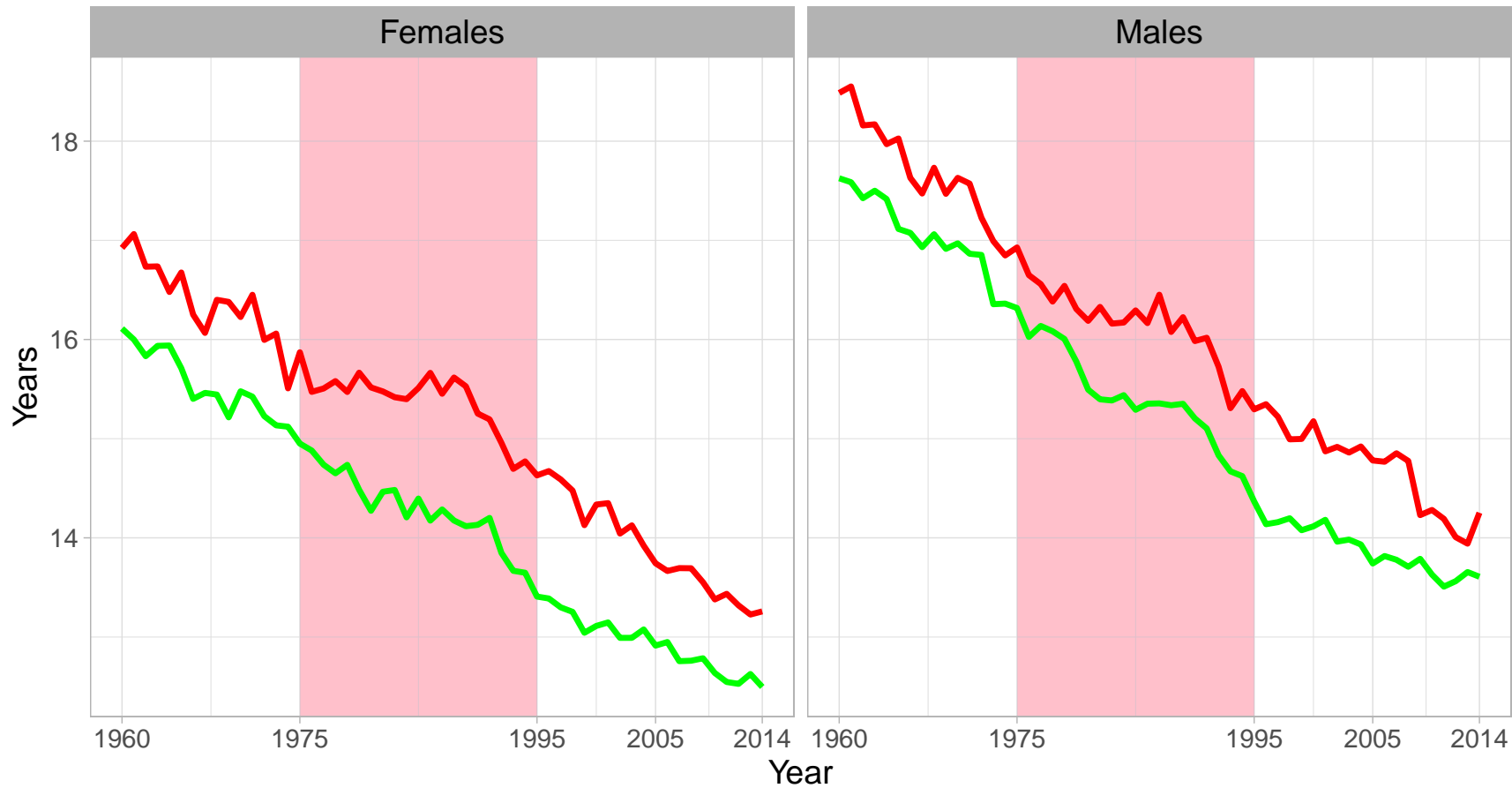

Supplement: Supplementary file 4 — Figure S3. Trends in the standard deviation for Sweden (green) and Denmark (red). (PDF 6 kb) [file 12889_2018_5730_MOESM4_ESM.pdf]
