# Supplementary material for: Potential gains in life expectancy by reducing inequality of lifespans in Denmark: an international comparison and cause-of-death analysis
Source: BMC Public Health. 2018 Jul 4;18:831. doi: 10.1186/s12889-018-5730-0 (PMC6033219; doi:10.1186/s12889-018-5730-0)
Supplement: Supplementary file 3 — Figure S1. Age and cause contributions to changes in life expectancy (panel A) and lifespan inequality (panel B) between 1960 and 1975, 1975–1995 and 1995–2014 for Danish males. Note: Age 0 is truncated in panel B since it accounts for the largest contribution. (PDF 126 kb) [file 12889_2018_5730_MOESM3_ESM.pdf]

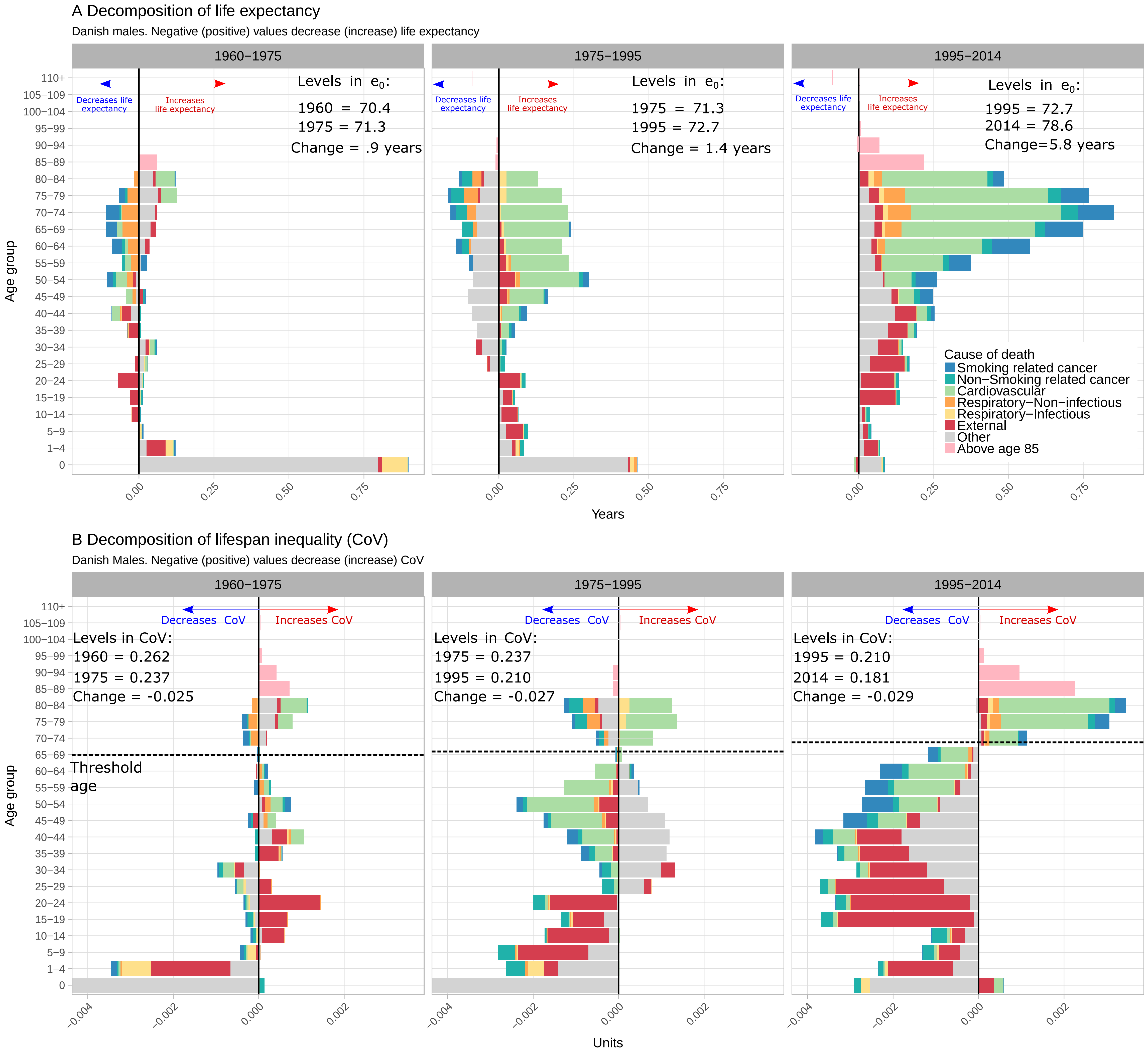
A Decomposition of life expectancy
Danish males. Negative (positive) values decrease (increase) life expectancy
1960−1975
1975−1995
1995−2014
Decreases life expectancy
Increases life expectancy
Levels in e_0:
1960 = 70.4
1975 = 71.3
Change = .9 years
Levels in e_0:
1975 = 71.3
1995 = 72.7
Change = 1.4 years
Levels in e_0:
1995 = 72.7
2014 = 78.6
Change=5.8 years
Cause of death
Smoking related cancer
Non−Smoking related cancer
Cardiovascular
Respiratory−Non−infectious
Respiratory−Infectious
External
Other
Above age 85
Age group
110+
105−109
100−104
95−99
90−94
85−89
80−84
75−79
70−74
65−69
60−64
55−59
50−54
45−49
40−44
35−39
30−34
25−29
20−24
15−19
10−14
5−9
1−4
0
0.00
0.25
0.50
0.75
Years
B Decomposition of lifespan inequality (CoV)
Danish Males. Negative (positive) values decrease (increase) CoV
1960−1975
1975−1995
1995−2014
Decreases CoV
Increases CoV
Levels in CoV:
1960 = 0.262
1975 = 0.237
Change = -0.025
Levels in CoV:
1975 = 0.237
1995 = 0.210
Change = -0.027
Levels in CoV:
1995 = 0.210
2014 = 0.181
Change = -0.029
Threshold age
Age group
−0.004
−0.002
0.000
0.002
Units
