# Supplementary material for: Potential gains in life expectancy by reducing inequality of lifespans in Denmark: an international comparison and cause-of-death analysis
Source: BMC Public Health. 2018 Jul 4;18:831. doi: 10.1186/s12889-018-5730-0 (PMC6033219; doi:10.1186/s12889-018-5730-0)
Supplement: Supplementary file 5 — Figure S4. Age and cause-decomposition of the change in the standard deviation over time for Danish females. Note: the age zero is truncated for visualization purposes. (PDF 9 kb) [file 12889_2018_5730_MOESM5_ESM.pdf]

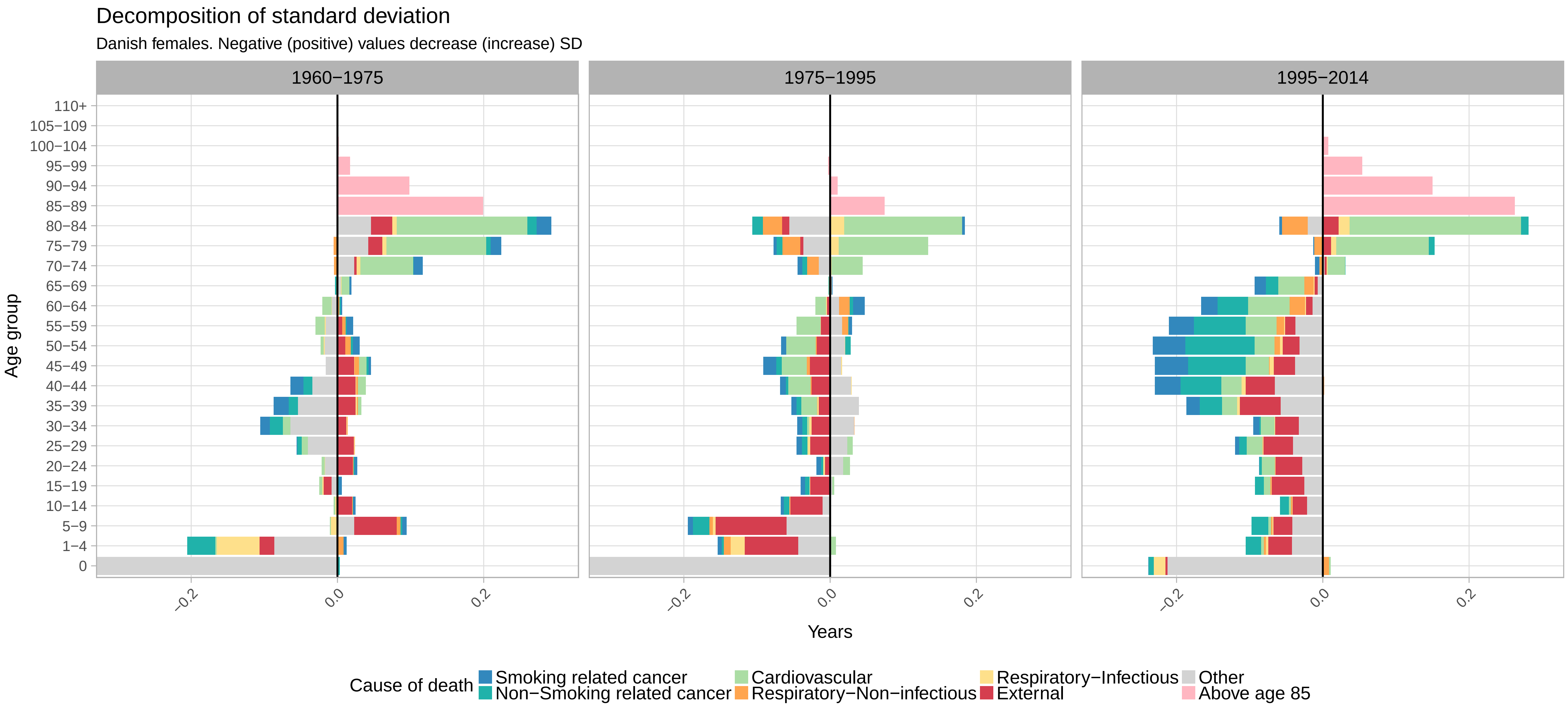
Decomposition of standard deviation
Danish females. Negative (positive) values decrease (increase) SD
1960−1975
1975−1995
1995−2014
Age group
110+
105−109
100−104
95−99
90−94
85−89
80−84
75−79
70−74
65−69
60−64
55−59
50−54
45−49
40−44
35−39
30−34
25−29
20−24
15−19
10−14
5−9
1−4
0
−0.2
0.0
0.2
Years
Cause of death
Smoking related cancer
Non−Smoking related cancer
Cardiovascular
Respiratory−Non−infectious
Respiratory−Infectious
External
Other
Above age 85
